# Latrophilin receptors: novel bronchodilator targets in asthma

**DOI:** 10.1136/thoraxjnl-2015-207236

**Published:** 2016-06-20

**Authors:** A Faiz, C Donovan, M AE Nieuwenhuis, M van den Berge, D S Postma, S Yao, C Y Park, R Hirsch, J J Fredberg, G Tjin, A J Halayko, K L Rempel, J P T Ward, T Lee, Y Bossé, D C Nickle, M Obeidat, Judith M Vonk, J L Black, B G Oliver, R Krishnan, B McParland, J E Bourke, J K Burgess

**Affiliations:** 1 Woolcock Institute of Medical Research, The University of Sydney, Glebe, New South Wales, Australia; 2 University of Groningen, University Medical Center Groningen, Department of Pulmonary Diseases, Groningen, The Netherlands; 3 Department of Pharmacology, Biomedicine Discovery Institute, Monash University, Clayton, Victoria, Australia; 4 Department of Pharmacology and Therapeutics, Lung Health Research Centre, University of Melbourne, Melbourne, Victoria, Australia; 5 Center for Vascular Biology Research, Department of Emergency Medicine, Beth Israel Deaconess Medical Center, Boston, Massachusetts, USA; 6 Program in Molecular and Integrative Physiological Sciences, Department of Environmental Health, Harvard T.H. Chan School of Public Health, Boston, Massachusetts, USA; 7 Manitoba Institute of Child Health, University of Manitoba, Winnipeg, Manitoba, Canada; 8 Kings College London, London, UK; 9 Department of Molecular Medicine, Institut universitaire de cardiologie et de pneumologie de Québec, Laval University, Québec, Quebec, Canada; 10 Merck Research Laboratories, Genetics and Pharmacogenomics, Boston, Massachusetts, USA; 11 Centre for Heart Lung Innovation, University of British Columbia, St. Paul's Hospital, Vancouver, British Columbia, Canada; 12 University of Groningen, University Medical Center Groningen, Department of Epidemiology, Groningen, The Netherlands; 13 Discipline of Pharmacology, Faculty of Medicine, The University of Sydney, Sydney, New South Wales, Australia; 14 School of Medical and Molecular Biosciences, University of Technology, Sydney, New South Wales, Australia; 15 University of Groningen, University Medical Center Groningen, Department of Pathology and Medical Biology, Groningen, The Netherlands

**Keywords:** Asthma, Asthma Genetics

## Abstract

**Background:**

Asthma affects 300 million people worldwide. In asthma, the major cause of morbidity and mortality is acute airway narrowing, due to airway smooth muscle (ASM) hypercontraction, associated with airway remodelling. However, little is known about the transcriptional differences between healthy and asthmatic ASM cells.

**Objectives:**

To investigate the transcriptional differences between asthmatic and healthy airway smooth muscle cells (ASMC) in culture and investigate the identified targets using in vitro and ex vivo techniques.

**Methods:**

Human asthmatic and healthy ASMC grown in culture were run on Affymetrix_Hugene_1.0_ST microarrays. Identified candidates were confirmed by PCR, and immunohistochemistry. Functional analysis was conducted using in vitro ASMC proliferation, attachment and contraction assays and ex vivo contraction of mouse airways.

**Results:**

We suggest a novel role for latrophilin (LPHN) receptors, finding increased expression on ASMC from asthmatics, compared with non-asthmatics in vivo and in vitro, suggesting a role in mediating airway function. A single nucleotide polymorphism in *LPHN1* was associated with asthma and with increased *LPHN1* expression in lung tissue. When activated, LPHNs regulated ASMC adhesion and proliferation in vitro, and promoted contraction of mouse airways and ASMC.

**Conclusions:**

Given the need for novel inhibitors of airway remodelling and bronchodilators in asthma, the LPHN family may represent promising novel targets for future dual therapeutic intervention.

**Key messages**

**What is the key question?**

- What is the specific role of latrophilins in airway smooth muscle cell phenotype?

**What is the bottom line?**

- This study found an increased expression of members of the latrophilin family of receptors in patients with asthma through comparison of healthy with asthmatic human airway smooth muscle (ASM) cells in culture, with the bottom line being that latrophilins may contribute to ASM contraction as well as adhesion and proliferation and their increased expression in asthmatic ASM may affect the asthma phenotype.

**Why read on?**

- This study provides novel insights into the transcriptional and functional differences between asthmatic and healthy ASM cells.

## Introduction

Asthma is a chronic inflammatory disease affecting 300 million people worldwide. With the incidence of asthma on the rise^1^ ^2^ and a proportion of patients remaining uncontrolled by current therapies,^3^ there is an unmet need to identify new therapeutic targets. In asthma, the major cause of morbidity and mortality is acute airway narrowing. This is in part the result of airway smooth muscle (ASM) hypercontraction, a feature specific to asthma.^4–6^ However, despite numerous studies focusing on the aberrant nature of asthmatic ASM cells, including increased proliferation, little is known about the receptors and pathways which may contribute to this hypercontractile phenotype.

Latrophilins (LPHNs) are a novel family of receptors previously thought to be brain specific,^7^ but with unknown physiological function.^8^ LPHNs are a unique class of G protein-coupled receptors, which can also act to facilitate cell adhesion.^9^ Originally identified for their ability to bind to α-latrotoxin (α-LTX), a toxin produced by the *Latrodectus* genus of spiders,^7^ they are best characterised for their role in the release of neurotransmitters (including acetylcholine (ACh)) from sensory and motor neurons, and also for the release of insulin from endocrine cells.

In this study, we compared gene expression profiles between asthmatic and healthy airway smooth muscle cells (ASMC) grown in vitro. Through this analysis, we identified the enhanced expression of *LPHN1* and *3* in asthmatic ASMCs. Using expression quantitative trait locus (eQTL) analysis in lung tissue, the expression of *LPHN1* was identified to be enhanced by a single nucleotide polymorphism (SNP) overrepresented in the asthmatic population. To demonstrate the role and function of the LPHN family in ASM, we tested known ligands and agonists in in vitro human cellular assays and ex vivo mouse airways. Ligands for LPHN receptors were able to directly and indirectly contract ASM and to contribute to the processes leading to remodelling of the airway wall present in asthma by promoting both ASM proliferation and attachment.

## Materials and methods

### ASMC isolation/culture and microarray analysis

Human ASMC were obtained from bronchial biopsies and explanted lungs from doctor diagnosed asthmatic patients (n=3) and healthy controls (n=3). ASMC were isolated and grown in culture as previously described.^10^

Total cellular mRNA was isolated using the Qiagen total RNA isolation kit (Qiagen, Doncaster, Victoria, Australia). Samples were labelled and run on an Affymetrix (Santa Clara, California, USA) GeneChip Human Gene 1.0 ST Arrays according to the manufacturer's instructions (GSE63383). Microarray analysis was conducted using R software V.3.02, using the Bioconductor-limma package, and normalised using Robust Multi-array Average.

### Validation of microarray results

The validation of the microarray results was undertaken at a transcriptional (quantitative real-time PCR) and translational (immunohistochemistry) level and the analyses are described in detail in the online supplementary materials.

10.1136/thoraxjnl-2015-207236.supp1 Supplementary data

### Single SNP and lung eQTL analysis

Single SNP analysis was conducted on polymorphisms within the *LPHN1* and *LPHN3* genes using the Dutch Asthma genome-wide association study (GWAS) (DAG) cohort, a cohort characterised by the presence of a doctor diagnosis for asthma and bronchial hyper-responsiveness.^11^ ^12^ Associations between *LPHN1* and *LPHN3* polymorphisms and the phenotype of asthma (defined by doctor diagnosis) and severity of bronchial hyperresponsiveness BHR of asthmatics (slope of BHR) were conducted.

SNPs significantly associated with risk of asthma were then explored in a large eQTL lung tissue dataset,^13^ to determine whether they played a functional role in LPHN gene expression. This lung eQTL dataset consisted of individuals with both genome-wide genotyping and microarray expression data from three sites of recruitment, University of Groningen, Laval University, University of British Columbia (UBC) (n=1095). Genome-wide gene expression and genotyping profiles were obtained using a custom Affymetrix array, Gene Expression Omnibus platform GPL10379 (GSE23546), and the Illumina Human1M-Duo BeadChip array, respectively. Standard quality controls were performed as described previously.^13^

### Human and animal tissue ethics

Isolated human airway tissue was obtained from explanted and resected lungs and post mortem organ donors with ethical approval from The University of Sydney and participating hospitals (Concord Repatriation General Hospital, Sydney South West Area Health Service and Royal Price Alfred Hospital). All patients provided written informed consent or, in the case of post mortem samples, consent was obtained from the next of kin. Demographic details of the patients used in this series of experiments are shown in online supplementary table S1. For cell contraction measurements, primary human ASMC were obtained with approval from the Gift of Hope Foundation, Illinois, USA. Approval for mouse tissue usage was through the University of Melbourne Animal Ethics Committee (approval number: #1212485). Non-tumour lung tissues were collected for the eQTL analysis from patients who underwent lung resection surgery at three participating sites: Laval University, University of Groningen and UBC.^13^

### Functional analysis

To identify the function of the LPHN receptors, the effects of the known LPHN ligands fibronectin leucine rich transmembrane protein 3 (FLRT3) and α-LTX were explored in immortalised airway smooth muscle cell (IASMC) proliferation (manual cell count) and attachment assays. To assess potential LPHN-induced contraction, the effects of FLRT3 and α-LTX on trachea and bronchi from Balb/C mice were measured ex vivo by myography. Measurements of cell contraction were conducted using contractile force screening.^14^ Briefly, cells were cultured to confluence on custom elastic substrates (polyacrylamide, stiffness=1.8 kPa) prepared within 96-well plates. In each well, the average contractile stress (in Pascal) was measured at baseline and following treatment(s). Data are reported as the force-response ratio, that is, the ratio of treatment to baseline contraction. Methods are described in detail in the online supplementary material.

### Densitometry and statistical analysis

Densitometric analyses of western immunoblots were conducted using Carestream Molecular Imaging Software using the mean intensity region of interest (ROI) for each band (Carestream Health, Rochester, New York, USA). For western immunoblot experiments, ROIs were compared with glyceraldehyde 3-phosphate dehydrogenase (GAPDH) in the same sample on the same membrane as an internal control. Statistical tests and graph plotting were conducted using GraphPad Prism 6 (GraphPad Software, La Jolla, California USA). A probability (p) value of <0.05 was considered statistically significant.

## Results

### Differential gene expression between asthmatic and healthy ASMC

**Figure 1:**
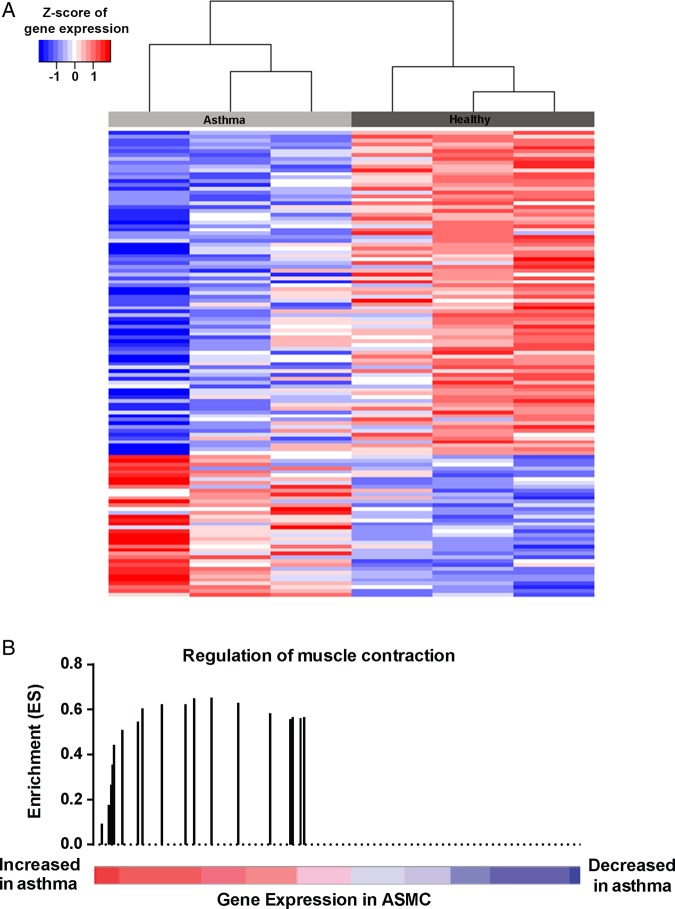
Microarray analysis of asthmatic and healthy airway smooth muscle cells (ASMC). (A) Hierarchy cluster analysis comparing asthmatic (n=3) and healthy (n=3) ASMC expression profiles with the rows identifying genes with fold change >±2, p<0.05. (B) Enrichment of genes involved in the regulation of muscle contraction associated with genes upregulated in asthmatic ASMC relative to healthy controls (GSEA false discovery rate (FDR) <0.05). The coloured bar represents genes ranked based on their differential expression between asthmatic and healthy ASMC. Vertical bars represent the running GSEA enrichment score and location (in the ranked gene list) of genes involved in the regulation of muscle contraction.

To evaluate differential expression between healthy and asthmatic ASMC, primary cells from healthy (n=3) and asthmatic (n=3) people were grown in vitro and the baseline expression profiles were measured using GeneChip Human Gene 1.0 ST Array; comparisons were conducted using limma statistical package for R (V.3.0.2). We identified 13 genes upregulated and 40 genes downregulated in asthmatic ASMC compared with healthy controls (false discovery rate (FDR) <0.5, fold change±2). The top 10 upregulated and downregulated genes are noted in online supplementary tables S2 and S3, respectively. A hierarchical cluster analysis is illustrated in figure 1A. Among the top genes enhanced in asthmatic ASMC were tropomyosin (*TPM1*), actin, gamma 2, smooth muscle, enteric (*ACTG2*) and *LPHN3.* While *TPM1* and *ACTG2* have been previously characterised for their roles in ASMC function, *LPHN3* was a newly identified candidate.

### Gene set enrichment analysis of genes differentially expressed in asthmatic ASMC

To identify key pathways that may be altered between asthmatic and healthy ASMC, gene set enrichment analysis (GSEA) analysis was conducted on the microarray dataset to extend the gene list. As biologically relevant to the cells studied, genes involved in the regulation of muscle and heart contraction were increased in asthmatic ASMC relative to healthy controls (GSEA FDR q<0.031) (figure 1B). An overview of all enriched pathways can be seen in online supplementary table S4.

### Asthmatic ASMC express higher levels of LPHN1 and 3

**Figure 2:**
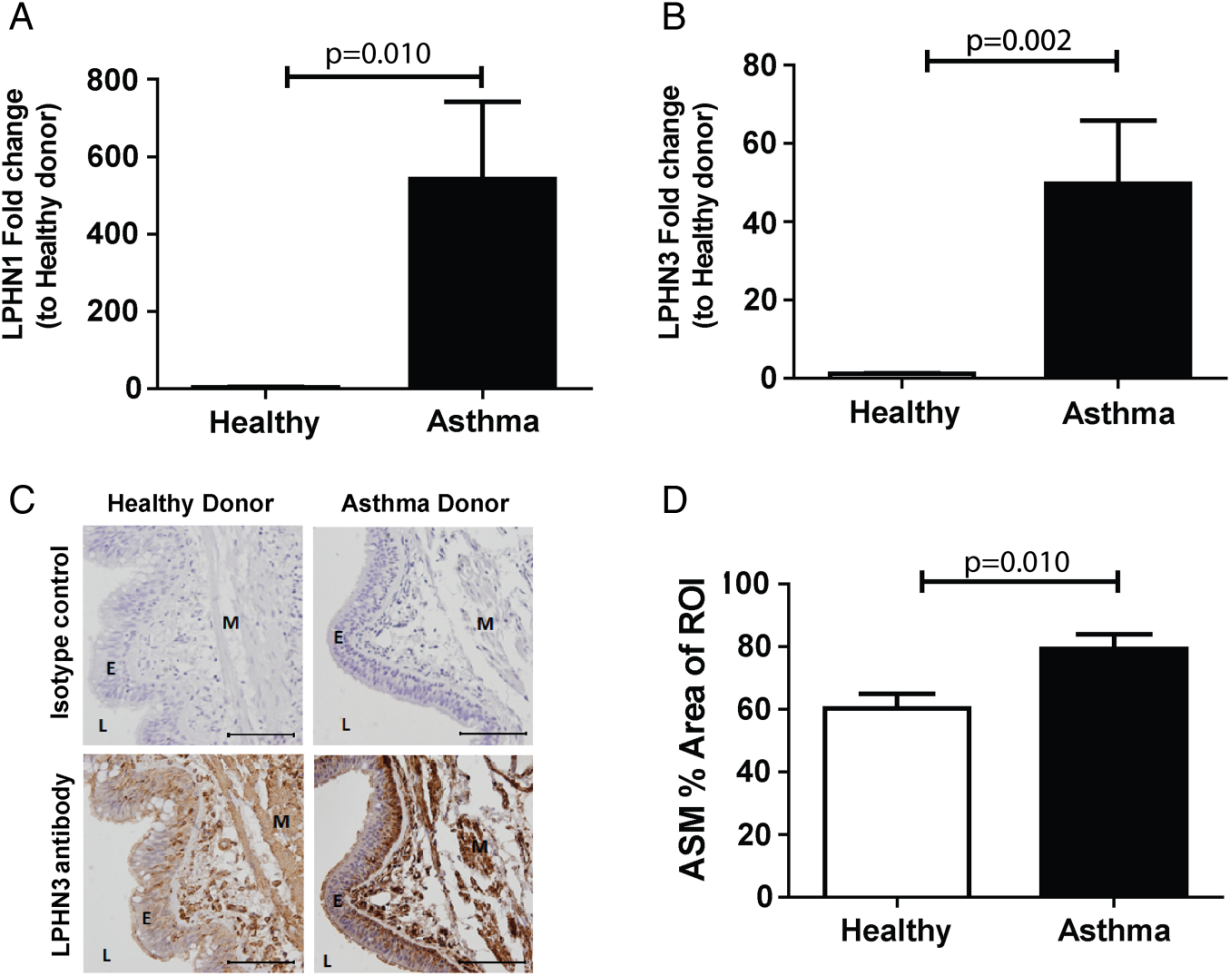
ASMC expression of latrophilins in cells from asthmatic and healthy airways. ASMC were grown to confluence in growth media (DMEM, 5% FBS and 1% antibiotics) and quiesced (DMEM, 0.1% BSA and 1% antibiotics) for 72 hours and total mRNA was extracted. qPCR of latrophilin family members LPHN1 (A) and 3 (B) comparing asthmatic (n=15) and healthy (n=6) ASMC. (C) Immunohistochemistry for LPHN3, comparing healthy (n=6) and asthma (n=11) donor bronchial sections (representative images). Specific staining was detected using a chemical chromophore 3,3'-diaminobenzidine (DAB) (brown) and cell nucleus was counterstained with haematoxylin (blue)(scale 100 μm). (D) ASMC-specific LPHN3 in healthy and asthma donor sections were quantified and compared using computerised image analysis. Data are expressed as mean±SEM. Statistical analysis used was Mann-Whitney test. ASMC, airway smooth muscle cell; BSA, bovine serum albumin; DMEM, Dulbecco's Modified Eagle Medium; E, epithelium; FBS, foetal bovine serum; L, lumen; M, airway smooth muscle; ROI, region of interest.

qPCR measurements of *LPHN* family members showed that mRNA levels of both *LPHN1* and *3* were significantly increased in asthmatic ASMCs compared with healthy (p=0.010 and p=0.002, respectively, Mann-Whitney test) (figure 2A, B), whereas no difference was found with *LPHN2* (data not shown). ASM-specific staining for *LPHN3* was detected, using immunohistochemical staining of bronchial biopsies, with cell density-independent elevation in patients with asthma compared with healthy controls (p=0.010, Mann-Whitney test) (figure 2C, D).

### Association of LPHN1 and LPHN3 SNPs with asthma

Two polymorphisms for *LPHN1* and 33 polymorphisms for *LPHN3* were available for analysis on the Illumina Chips, and associations with the asthma phenotype were investigated. A single SNP in *LPHN1* (rs3810256) was identified, with the minor allele increasing the risk of asthma (FDR=0.016) (table 1). Associations between *LPHN1* and *LPHN3* polymorphisms and severity of bronchial hyper-responsiveness in asthmatics were conducted in the DAG cohort; however, no associations were found (see online supplementary table S5).

**Table 1:** SNPs for LPHN1 and LPHN3 associations with asthma

CHR	BP	SNP	TA	OR	OR(R)	P	P(R)	I	FDR
LPHN1									
19	14120846	**rs3810256**	A	1.348	1.348	0.008	0.008	0	0.016
19	14159442	rs2420416	G	1.032	1.032	0.676	0.676	0	1
LPHN3									
4	62327627	rs12509742	A	0.986	0.986	0.829	0.829	0	1
4	62330503	rs2345043	T	0.982	0.982	0.788	0.788	0	1
4	62352873	rs1450903	G	0.848	0.846	0.095	0.356	70.21	1
4	62354238	rs10517547	A	0.962	0.960	0.665	0.808	71.98	1
4	62376287	rs11734607	T	1.010	1.009	0.895	0.917	29.69	1
4	62380952	rs2345041	A	1.083	1.083	0.364	0.364	0	1
4	62401922	rs2015569	T	0.899	0.898	0.297	0.508	60.12	1
4	62409295	rs7667328	G	0.978	0.983	0.839	0.934	71.67	1
4	62409469	rs10446786	G	1.008	1.007	0.915	0.932	24.10	1
4	62416032	rs13110933	C	1.069	1.069	0.314	0.314	0	1
4	62422136	rs6551665	G	0.907	0.907	0.150	0.272	41.51	1
4	62422269	rs6846033	C	0.980	0.980	0.801	0.801	0	1
4	62430979	rs9683662	T	1.029	1.029	0.691	0.691	0	1
4	62436915	rs6858066	G	1.039	1.039	0.563	0.621	28.83	1
4	62441865	rs11131347	T	0.928	0.928	0.246	0.246	0	1
4	62444465	rs1470724	C	1.093	1.093	0.227	0.227	0	1
4	62446070	rs6551666	C	1.000	1.002	0.999	0.990	60.75	1
4	62471863	rs10517549	G	1.016	1.016	0.831	0.831	0	1
4	62483323	rs734644	T	1.027	1.027	0.729	0.729	0	1
4	62499334	rs1450896	A	0.776	0.775	0.182	0.318	44.74	1
4	62501063	rs995447	C	1.046	1.046	0.729	0.729	0	1
4	62513128	rs1510925	G	1.062	1.064	0.473	0.601	50.00	1
4	62517697	rs1397545	A	1.052	1.052	0.646	0.646	0	1
4	62521660	rs1397543	T	1.041	1.043	0.633	0.724	50.03	1
4	62528085	rs1397548	A	1.062	1.062	0.416	0.416	0	1
4	62533752	**rs17292128**	C	1.207	1.207	0.034	0.034	0	1
4	62539254	rs10017760	A	1.023	1.023	0.797	0.797	0	1
4	62544980	rs2271339	G	1.073	1.072	0.352	0.547	56.20	1
4	62550048	rs13115125	G	1.044	1.043	0.511	0.765	78.09	1
4	62566026	rs1510920	C	0.852	0.839	0.281	0.479	63.93	1
4	62584757	rs6827266	T	0.950	0.950	0.431	0.431	0	1
4	62597619	rs1397546	C	0.918	0.914	0.198	0.554	80.54	1
4	62606392	rs11736888	T	1.016	1.016	0.839	0.839	0	1

BP, base pair; CHR, chromosome; FDR, false discovery rate; I, heterogeneity of samples; LPNH, latrophilin; R, random effect; SNP, single nucleotide polymorphism; TA, tested allele bold indicates SNPs associated with asthma.

### rs3810256 polymorphism is associated with LPHN1 gene expression

To further quantify the role of the rs3810256 SNP on *LPHN1* gene expression, eQTL meta-analysis on three cohorts was conducted to determine if this SNP was associated with mRNA expression levels of *LPHN1* in human lung tissues. The eQTL analysis identified a single probe within *LPHN1* which had increased expression associated with the rs3810256 minor allele (identified to increase the risk of asthma) in the Laval cohort (β±SE=0.08±0.02, p=0.001), and a trend towards association was seen in the Groningen cohort (β±SE=0.05±0.03, p=0.16) and the UBC cohort (β±SE=0.04±0.03, p=0.18) (table 2). Meta-analysis of all three cohorts identified an overall significant effect of the rs3810256 minor allele on increased expression of the *LPHN1* gene (β±SE=0.06±0.02, p<0.001).

**Table 2:** eQTL analysis of rs3010256 in LPHN1

Probe	SNP	TA	(ref 36.3) Start probe	(ref 36.3) End probe	Cohort	β	SE	p Value
*LPHN1*	rs3810256	A	14119550	14177997	Groningen	0.048	0.034	0.160
					Laval	0.076	0.023	1.000E-03
					UBC	0.038	0.028	0.175
					*Meta*	0.058	0.016	2.330E-04

eQTL, expression QTL; LPNH, latrophilin; meta, combined analysis of Groningen, Laval and UBC cohorts; SNP, single nucleotide polymorphism; TA, tested allele; UBC, University of British Columbia.

### FLRT3 promoted adhesion and proliferation of asthmatic ASMC via LPHN3

**Figure 3:**
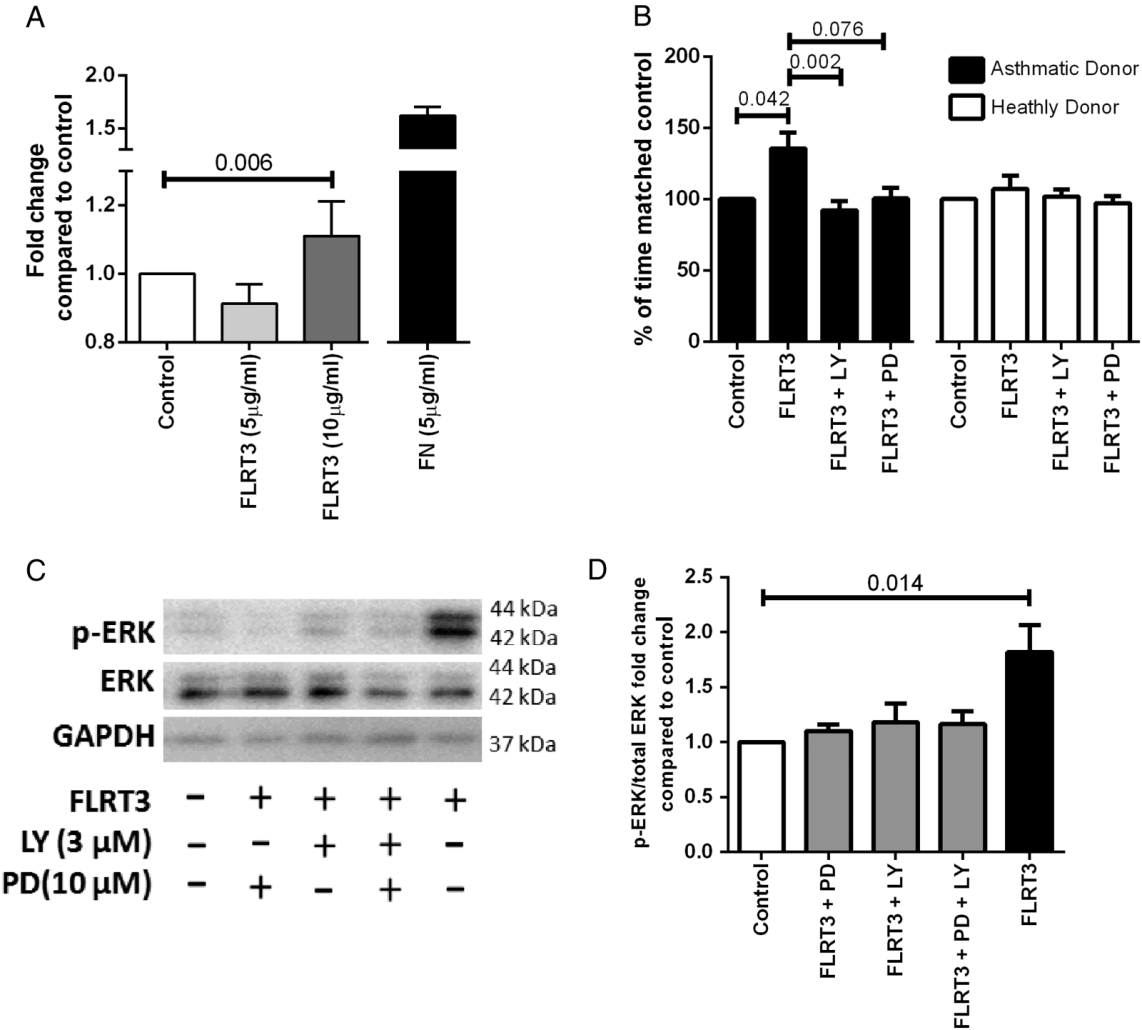
FLRT3 effect on IASMC attachment and proliferation in asthma and healthy donor cells. (A) IASMC were grown to confluence in growth medium (DMEM, 5% FBS and 1% antibiotics) and quiesced (DMEM, 0.1% BSA and 1% antibiotics) for 24 hours, seeded at 16 000 ASMC per well in quiescing media and left for 2 hours at 37°C in 5% CO_2_ (n=7). Attached cells were stained with toluidine blue measured by spectrophotometry at absorbance 595 nm. Data are expressed as mean±SEM. Friedman test, Dunn's correction was used to compare control to other treatments. IASMC proliferation was measured by manual cell count following quiescing for 72 hours and then treatment with (B) quiescing medium (control), FLRT3 (10 ng/mL), FLRT3 (10 ng/mL)+PD98059 (10 μM) and FLRT3 (10 ng/mL) +LY294002 (3 μM) for 72 hours (asthmatics, n=6 and healthy donors, n=6). Data are expressed as mean±SEM. Friedman test, Dunn's correction was used to compare (#=p<0.05 compared with control, ‡=p<0.05 compared with FLRT3). (C) ASMC were seeded and grown to confluency and quiesced for 72 hours and then treated with quiescing medium (control), FLRT3 (10 ng/mL), FLRT3 (10 ng/mL)+PD98059 (10 μM) and FLRT3 (10 ng/mL)+LY294002 (3 μM). (D) Representative western immunoblot for pERK1/2, total ERK1/2 and GAPDH H (n=8) (D) Densitometric analysis of ERK1/2 western. Data are expressed as mean±SEM. Friedman test, Dunn's correction was used to compare. ANOVA, analysis of variance; ASMC, airway smooth muscle cells; BSA, bovine serum albumin; DMEM=Dulbecco's Modified Eagle Medium; ERK1/2, extracellular signal-regulated protein kinases 1 and 2; FBS, foetal bovine serum; FLRT3, fibronectin leucine rich transmembrane protein 3; GAPDH, glyceraldehyde 3-phosphate dehydrogenase; IASMC, immortalised airway smooth muscle cells; LY, LY294002; PD, PD98059; pERK1/2, phosphorylated extracellular signal-regulated protein kinases 1 and 2.

The functional role of LPHN3 in ASMC was investigated using its known endogenous ligand, FLRT3. FLRT3 (10 ng/mL) enhanced adhesion of IASMC cells (p=0.037, Friedman test, Dunn's correction) and specifically enhanced proliferation of asthmatic IASMC cells (p=0.042, Friedman test, Dunn's correction) (figure 3A, B). Proliferation was blocked in asthmatic IASMC by either the phosphoinositide 3 kinase inhibitor, LY294002 (3 μM), or a trend towards significance with the mitogen-activated protein kinase kinase (MEKK) inhibitor, PD98059 (10 μM) (p=0.002 and p=0.076, respectively, Friedman test, Dunn's correction) (figure 3B), which blunted extracellular regulated kinase (ERK) phosphorylation (figure 3C, D).

### Activation of LPHN1 and 3 caused contraction in mouse airway preparations

Mouse tracheal and bronchial tissues were mounted on myographs at an optimum length for maximal force generation in response to the potassium depolarising solution (KPSS). Thereafter, the mean active force generated during two contractions induced by ACh 10^−4^ M was determined; 7.2±0.7 and 6.1±0.7 mN for trachea (n=15) and bronchi (n=12), respectively. Only tissues responding to both KPSS and ACh were included. These responses were used to normalise contractile responses induced via LPHN receptors.

**Figure 4:**
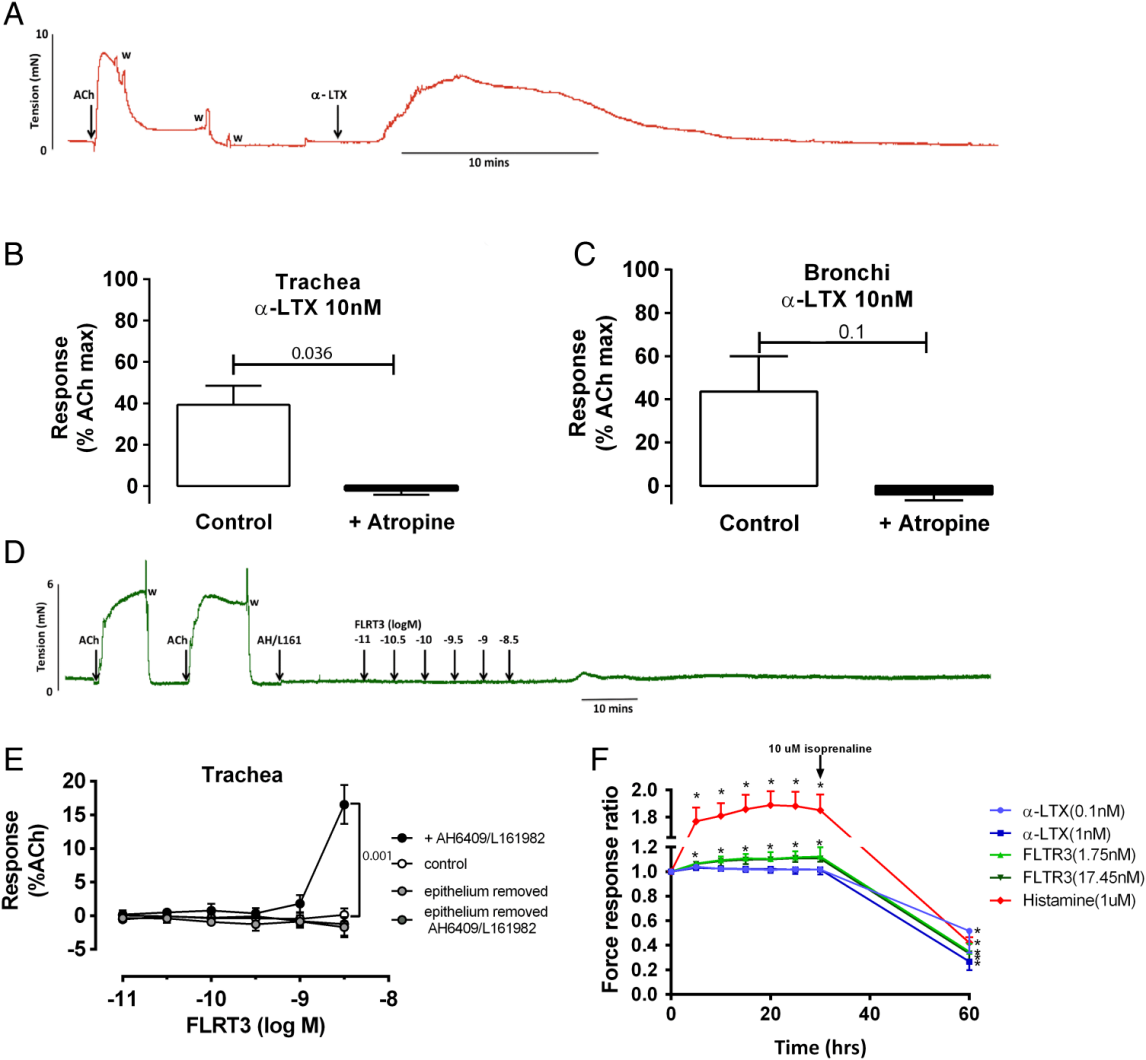
The effect of α-LTX and FLRT3 on mouse tracheal and bronchial preparations and airway smooth muscle cells (ASMC) contraction assays Male Balb/C mice were killed by intraperitoneal injection of 0.4 mL sodium pentobarbitone (60 mg/mL) at 6–8 weeks of age. After an equilibration period of 30 min, tissues were contracted to KPSS at differing tensions (1.2, 1.5, 1.8 mN for trachea; 1.0, 2.0, 3.0 mN for bronchi) to determine the optimal resting tension for each tissue that corresponded to the highest KPSS response. Tissues were then contracted to ACh (10^−4^ M) twice to determine the effect of maximum muscarinic receptor activation. α-LTX- (10 nM) induced contraction of trachea (A and C) and bronchi (D) in the absence (white bars) and presence (black bars) of atropine (3 μM), n=3–5. B and E) Concentration-dependent effect of FLRT3 (10–3000 pM) in mouse trachea in the presence (open circles, n=7) or absence of epithelium (light grey circles, n=4) or EP2 and EP4 antagonists (AH6809 3 μM and L-161982 1 μM) in the presence (black circles, n=6) or absence of epithelium (dark grey circles, n=4). Statistical analysis used was Mann-Whitney test. (F) Single cell contraction assay of ASMC treated with LTX (0.1, 1 nM), FLRT3 (1.75, 17.45 nM) or histamine (1 µM) for 30 min and then relaxed with isoprenaline (10 µM) (n=10). Statistical analysis used was Mann-Whitney test. All data are expressed as mean±SEM. Ach, acetylcholine; α-LTX, α latrotoxin, FLRT3, fibronectin leucine rich transmembrane protein 3.

Exposure of trachea or bronchi to α-LTX elicited ∼40% of the ACh maximum response in these tissues (figure 4A–C). Both the ACh and α-LTX-induced contractions were blocked by the muscarinic antagonist atropine (3 μM, p=0.036, Mann-Whitney test, figure 4B, C).

At low concentrations (10^−11^–10^−9.5^ M), the LPHN3 ligand, FLRT3, had no effect on basal tone in isolated tracheal preparations with or without epithelium (control, figure 4E). However, FLRT3 elicited contraction at the highest concentrations tested (10^−8.5^ M) in intact trachea in the presence of EP2/4 antagonists (+ AH6704/L161982) (figure 4D, E). This contraction to FLRT3 was abolished when the epithelium was removed (figure 4E).

### Activation of LPHN3 but not LPHN1 caused contraction in human ASMC

The direct effects of α-LTX and FLRT3 were assessed using the experimental approach of contractile force screening.^14^ The baseline force was used to normalise contractile responses induced by α-LTX, FLRT3 and histamine. Exposure of ASMC to FLRT3 (1.75 and 17.45 nM) induced a sustained contraction for 30 min (p<0.05), which was then relaxed with the addition of isoprenaline. In contrast, exposure to α-LTX (0.1 and 1 nM) did not induce contraction (p>0.05). α-LTX (10 nM) had a cytotoxic effect on ASMC under subconfluent conditions and was unable to be run in this cellular model.

## Discussion

In this study, genome-wide gene expression was used as a hypothesis generating method to identify genes differentially expressed between asthmatic and healthy ASMC. The gene expression profile from the subsequent analysis differed between the asthmatic and healthy phenotypes, with genes associated with muscle contraction being enhanced in asthmatic ASMC. The gene candidate *LPHN3* identified in the microarray analysis was verified in a larger patient group at both the transcriptional and translational levels. The expression levels of the other LPHN family members were measured, with *LPHN1* also increased in asthmatic ASMC at the transcriptional level. A single SNP (rs3810256) in *LPHN1* was found to associate with an increased risk of asthma. Lung eQTL analysis identified that the rs3810256 SNP minor allele was associated with increased expression of *LPHN1,* this direction of effect is consistent with *LPHN1* upregulation in asthmatics.

The function of the LPHN1 and 3 receptors were explored using a known agonist α-LTX and an endogenous LPHN3 ligand FLRT3. FLRT3 facilitated adhesion and proliferation of ASMC with the latter being enhanced only in patients with asthma. Stimulation with α-LTX contracted tracheal and bronchial tissues from mice; however, this was found to be an indirect response mediated through the release of ACh as indicated by blocking the response with the muscarinic antagonist, atropine. FLRT3 caused contraction of both trachea in the presence of prostaglandin receptor (subtypes EP2/4) antagonists and ASMC in contraction assays conducted in the absence of epithelium.

GSEA analysis identified that genes which regulate muscle contraction were overrepresented in the grouping of genes overexpressed in asthmatic ASMC relative to healthy controls. The literature is currently divided on whether the hypercontractility seen in asthmatic ASMC has an environmental or intrinsic origin. However, this finding may begin to elucidate the mechanism underlying the increased contraction of asthmatic ASMC to contractile agents relative to healthy controls found in some studies independent of environmental influences that promote BHR, perhaps driven by genetic or epigenetic factors.^15^ ^16^

ASMC from patients with asthma were found to have an increased rate of attachment to FLRT3. The FLRT3-LPHN3 interaction may assist the docking of other cell types expressing FLRT3 on their surfaces to ASMC, a mechanism that is augmented in the asthmatic airway. Such a system has been reported in cell lines overexpressing LPHN3 and FLRT3.^9^ Previously the expression of *FLRT3* has been shown to be upregulated following stimulation with lipopolysaccharide and by *Legionella pneumophila* infections in a number of inflammatory cells.^17–19^ Therefore, it is tempting to postulate that the increased presence of LPHN3 receptors on asthmatic ASMC may enhance the docking of inflammatory cells to ASMC during inflammatory episodes.

FLRT3 induced proliferation of asthmatic ASMC (∼142%) is comparable with the induction of proliferation by platelet-derived growth factor subunit B (PDGF-BB) (∼150%) (a well-known ASMC proliferation stimulus)^20^ following 72H treatment in serum-reduced medium. This proliferation of asthmatic ASMC was blocked following the inhibition of the mitogen-activated protein kinase (MAPK) pathway. As the activation of the MAPK pathway, through ERK1/2 phosphorylation, is enhanced in asthmatic ASMC,^21^ it is therefore probable that the FLRT3-LPHN3 interaction contributes to enhanced proliferation of asthmatic ASMC.^22^ ^23^ These results suggest that LPHN3 may be a potential target for the regulation of remodelling in the asthmatic airway.

In previous studies in non-airway tissues, α-LTX induced release of ACh, a potent contractile agent of ASM;^24–26^ in skeletal muscle preparations, ACh release from small synaptic vesicles in response to α-LTX led to contraction.^26^ ^27^ Since the response to both ACh and α-LTX in the current study was ablated by the muscarinic antagonist, it appears that α-LTX mediated airway contraction through the release of Ach by cholinergic nerves^28^ and/or the epithelium,^29^ rather than by the direct activation of LPHN1 on the ASMC. This was further highlighted with the lack of contraction of ASMC to α-LTX in the cell contraction assay which was conducted in the absence of cholinergic nerves and the epithelium.

The effects of α-LTX on airway contraction, reported here for the first time, provide pivotal experimental evidence elucidating the mechanism underlying the breathing difficulties, including bronchospasm and chest tightness, associated with bites from the widow genus spiders.^30^ Furthermore, these data support the use of atropine in addition to traditional anti-venom therapy to treat airway distress associated with widow genus spider bites.

Selective activation of LPHN3 by FLRT3 in mouse trachea also resulted in contraction. However, this was only evident in trachea with an intact epithelium, and when the potential contribution of prostaglandin E2 (PGE_2_), an endogenous mediator that promotes airway relaxation^31^ was also inhibited. Since a consistent contractile response to FLRT3 was obtained in tissues treated with EP2/EP4 antagonists at concentrations previously shown to inhibit PGE_2_-mediated relaxation in mouse intrapulmonary airways,^32^ it appears that FLRT3-mediated contraction may be physiologically opposed by endogenous PGE_2_ promoting relaxation. The lack of response to FLRT3 following epithelial removal under these conditions suggests that it may facilitate the release of contractile factors from the epithelium leading to contraction of ASM. In contrast to these findings in mouse airways, FLRT3 induced ASMC contraction in the cell contraction assay despite the absence of the epithelium. Together these results suggest that FLRT3 promotes contraction indirectly via the epithelium in mouse trachea and directly in human ASMC.

Since FLRT3 represents the endogenous ligand for LPHN3, the effects observed in the present study may have physiological implications with respect to asthma in terms of increasing bronchial tone. This will need to be investigated further using selective antagonists to the LPHN receptors. Given that the relaxation signalling cascade may be impaired in asthma, activation of the LPHN3 receptor may account in part for the increase in airway contractility in asthma.

The main strength of this study was the multidisciplinary approach used to identify a novel gene target using mass screen approaches including both microarray analysis and candidate SNP/eQTL analysis followed by the functional interrogation of the candidate using in vitro/ex vivo models. There were some limitations to this study that should be acknowledged. First, the low numbers of subjects screened and the less stringent FDR in the initial microarray analysis. The impact of this limitation was overcome by the qPCR verification of the identified candidates in an expanded group of asthmatic and healthy ASMC. Second, as the microarray analysis was conducted in vitro it is possible that the gene expression differences may be due to senescence or differences in the rate of proliferation and dedifferentiation in culture. However, to limit this effect all patient cells included in the microarray arrays were grown under the same conditions and at the same passage number. Third, as both LPHN1 and LPHN2 have the ability to interact with α-LTX, the effect of LPHN2 on the ASMC was unable to be excluded as a factor which may have contributed to the in vitro functional analysis. Finally, the rs3810256 risk allele was identified to be significantly associated with increased expression of *LPHN1* in the meta-analysis of all three cohorts; individual analysis of the UBC and Groningen cohorts was not found to be significant, yet the directions of the effects were the same. One of the possible reasons for this finding may have been the source of the lung tissue used for expression analysis. The UBC and Groningen cohorts were considerably more heterogeneous (wider variety of lung diseases) than the Laval cohort. As the amount of ASMC in each patient can vary significantly depending on the underlying disease phenotype, this may have affected the level of LPHN1 mRNA detected irrespective of the SNP genotype.

This study identified for the first time, to our knowledge, the expression of *LPHN1* and *LPHN3* in ASMC, and found these to be enhanced in patients with asthma. The differential effects of FLRT3 in asthmatic and healthy airways may potentiate both airway remodelling and contraction in asthma. Functional analyses suggest a modest role of LPHN receptors in ASM adherence and proliferation. A similar modest effect of direct activation of ASM LPHN receptors on ASM cell contractility was observed, although a more robust effect of LPHN receptor antagonism was noted in ASM tissue, likely due to effects on epithelia and neurotransmission. The airway contraction observed in the presence of α-LTX, mediated through the release of ACh, identified the mechanism underlying bronchial spasms and chest tightness associated with bites from the widow genus spiders. On the basis of these findings, we suggest that blocking of the LPHN receptors in the airways may provide novel targets for future therapeutics for asthma.
