# Supplementary material for: Latrophilin receptors: novel bronchodilator targets in asthma
Source: Thorax. 2016 Jun 20;72(1):74–82. doi: 10.1136/thoraxjnl-2015-207236 (PMC5329048; doi:10.1136/thoraxjnl-2015-207236)
Supplement: Supplementary data [file thoraxjnl-2015-207236supp.pdf]

Supplementary Materials

Airway smooth muscle culture

Following isolation and initial growth, ASMC at passages 3-4 were seeded at $1x10^6$ cell/cm^2 and grown in growth media (Dulbecco's modification of Eagle's medium (DMEM) (Invitrogen), 5% foetal bovine serum (FBS) and 1% antibiotics (Invitrogen)) for 3-4 days. Cells were then quiesced in DMEM supplemented with 0.1% bovine serum albumin (BSA) (Sigma Aldrich, St Louis, MO, USA), and 1% antibiotics (Invitrogen)) for 72 hours.

Candidate gene analysis of LPHN1&3

This study was approved by the medical ethical committee and consistent with the Research Code of the University Medical Center Groningen and Dutch national ethical and professional guidelines ("Code of conduct; Dutch federation of biomedical scientific societies"; **http://www.federa.org**). Candidate gene analysis was conducted on SNPs within 200kb flanking the *LPHN1* and *3* genes using the Dutch Asthma GWAS (DAG) cohort. The DAG cohort consists of 920 asthma cases and 980 controls, all from the northern part of the Netherlands. This cohort was genotyped in two phases and meta-analysed afterwards. For the first phase, 468 cases were selected from a trio and family study. The 469 controls were non-asthmatic spouses or pseudo-controls of untransmitted alleles in our trio design (GWAS I) . For the second phase (GWAS II), 452 asthmatics were selected from previous clinical and genetic studies performed by our research institute. The 511 controls were selected from the COPACETIC study, a geographically matched population-based study on lung cancer screening in male smokers [1].

All asthmatics had asthma symptoms, a physician's diagnosis of asthma, , and BHR to either histamine or methacholine. Controls had no asthma or COPD, nor any evidence of significant airway obstruction. BHR was measured with a methacholine or histamine challenge test, and defined as PC_{20} histamine.

The severity of BHR was calculated based on the slope of the challenge test with either methacholine or histamine. The slope was calculated by dividing the difference between FEV_1 at baseline and at the dose step at which a 20% fall or more in FEV_1 was reached, by the dose that was inhaled at this last step. We divided the BHR slopes of the 30-second tidal breathing method by 4 in order to compare the slope of the 30-second tidal breathing method with the 2-minute method. Values were log transformed to reach normal distribution.
650 Asthmatics with measurements on ICS use and smoking history were included in the analysis. Linear regression analyses were performed in an additive model to analyze the effect of SNPs on slopes of the BHR test with adjustments for smoking and inhaled and/or oral corticosteroid use. Smoking was labelled as never, current or ex-smokers. ICS use was labelled as the time stopped before the challenge test: 1) never on steroids or stopped longer than 4 weeks, 2) stopped between 2-4 weeks or 3) stopped between 0-2 weeks before the challenge test.

Expression QTL analysis

Ethics approval

At Laval, lung specimens were collected from patients undergoing lung cancer surgery and stored at the "Institut universitaire de cardiologie et de pneumologie de Québec" (IUCPQ) site of the Respiratory Health Network Tissue Bank of the "Fonds de recherche du Québec – Santé" (www.tissuebank.ca). Written informed consent was obtained from all subjects and the study was approved by the IUCPQ ethics committee. At Groningen, lung specimens were provided by the local tissue bank of the Department of Pathology and the study protocol was consistent with the Research Code of the University Medical Center Groningen and Dutch national ethical and professional guidelines ("Code of conduct; Dutch federation of biomedical scientific societies"; http://www.federa.org). At Vancouver, the lung specimens were provided by the James Hogg Research Center Biobank at St Paul's Hospital and subjects provided written informed consent. The study was approved by the ethics committees at the UBC-Providence Health Care Research Institute Ethics Board.

eQTL analysis

Probes located in the genes of interest were selected for *cis*-eQTL analysis (genome build reference 36.3). eQTL analyses were performed per site and then meta-analyzed. To correct for technical variance, principal components were calculated on the residuals of a linear regression on the gene-expression data with correction for gender, age and smoking. Principal components which explained more than 5% of the variance were added as covariates in the eQTL analysis on the gene-expression data. eQTL analysis was performed using a linear regression with correction for gender, age, smoking and technical variance (principal components). Selected probes were checked for any known polymorphisms in the probe (primer polymorphisms).

Quantitative real-time PCR (qPCR)

To validate microarray results, qPCR was conducted on total mRNA isolated from asthmatic (n=15) and healthy (n=6) ASMC cultured using the same method as microarray samples. qPCR was conducted using Taqman primers (LPHN1(Hs00208706_m1), 2 (Hs00202347_m1) and 3(Hs00248624_m1)), and was normalised to 18S (4319413E)(Life Technologies). Mann whitney U t-test were used to compare LPHN gene expression levels between asthmatic and healthy ASMC.

ASMC hTERT Immortalisation

Immortalised ASMCs (IASMC) were used to confirm findings from primary ASMC and identify the functional role of LPHN family of receptors in asthmatic and healthy IASMC lines. The immortalisation of primary ASMC was carried out as described previously [2].

Immunohistochemistry

To measure expression of LPHN3, *ex vivo* immunohistochemistry was conducted on airway bronchial sections using rabbit anti-human LPHN3 antibody [500 ng/ml], which was performed in parallel with rabbit IgG (DakoCytomation) as an isotype control at the same concentration as the primary antibody, according to Faiz *et al.* (2013). Quantification of immunostaining of the images taken was completed using Image J (v1.42q, NIH) to define the area of staining (the number of brown pixels in the image). Area of interest analysis was used for the evaluation of ASM specific immunostaining of LPHN3 which was determined for 5 representative images of each section (patient) and averaged. Mann whitney U t-test were used to compare LPHN3 protein levels between asthmatic and healthy sections.

Attachment assay

To investigate the function of the LPHN3 in cellular attachment to FLRT3 attachment assays were conducted. Briefly, 96 well plates were coated with FLRT3 (5 and 10 ng/ml) or fibronectin (5 ng/ml) in PBS for 2 hours at 37°C. The plate was then blocked with 1% BSA in PBS for 1 hour at 37°C. 16,000 ASM cells per well were seeded in *quiesc*ing media and left for 2 hours at 37°C in 5% CO_2. Unattached cells were removed by washing 3 times with PBS. Cells were then fixed with 1% (v/v) paraformaldehyde for 5 minutes and stained with ice cold (0.5%) toluidine blue (Sigma-Aldrich St. Louis, MO, USA) containing 0.5% (v/v) boric acid (Sigma-Aldrich) for an additional 5 minutes. Stained cells were washed with distilled water and solubilized using 1% (w/v) Sodium dodecyl sulphate (SDS) in PBS. Cell attachment was measured by spectrophotometry at absorbance 595 nm using the Spectramax M2 (Molecular Devices, Sunnyvale, CA, USA) and collected using Spectramax software (Molecular Devices). Friedman test with Dunn’s correction was used.

Proliferation assay

To investigate the function of FLRT3 in ASMC proliferation a proliferation assay was conducted. Briefly, ASMC were seeded at $5x10^3$ cells/cm^2 overnight in growth media (DMEM, 10% FBS 1% antibiotics) ASMC before being *quiesced* for 72 hours. Cells were then treated with either *quiesc*ing media (control), FLRT3 (10 ng/ml) or α-LTX (1 nM), FLRT3 (10 ng/ml) or α-LTX (1 nM) + PD98059 (10 μM) or FLRT3 (10 ng/ml) or α-LTX (1

nM) +LY294002 (3 μM) for 72 hours. Proliferation was measured by manual cell counts as previously described [3]. Friedman test with Dunn's correction was used.

Myographs

Trachea and bronchi were isolated from male Balb/C mice were prepared as previously described [4].

Briefly, male Balb/C mice were killed by intraperitoneal injection of 0.4 mL sodium pentobarbitone (60 mg/mL) at 6-8 weeks of age. Bronchial smooth muscle reactivity was analysed in temperature-controlled (37°C) myographs (Organ Bath Model 700MO, J.P. Trading, Aarhus, Denmark) containing KREBS solution continuously equilibrated with 5% CO_2 and 95% O_2. Fine wire (50 μm) was threaded through the tissue lumen and connected to a force displacement transducer and micrometer to record changes in isometric tension (ΔmN) via Power Lab and Chart software (AD Instruments Ltd, Hastings, U.K.). After an equilibration period of 30 min, tissues were exposed to KPSS at differing tensions (1.2, 1.5, 1.8mN for trachea; 1.0, 2.0, 3.0 mN for bronchi) in order to determine an optimal resting tension for each tissue, corresponding to the highest KPSS response. Tissues were then contracted with ACh 10^{-4} M and treated with either α-LTX or FLRT3. For α-LTX treatments tissues were pre-treated with or without atropine (3 μM) for 20 min and then treated with α-LTX (10 nM). FLRT3 was added cumulatively (10-3000 pM) in the presence and absence of the epithelial layer or in the presence of EP2/4 antagonists (AH6809 (3 μM) and L161982 (1 μM)). The FLRT3 was then washed out and bronchi pre-contracted to ACh and then treated with substance P (SP) to confirm the presence (relaxation) or absence (no relaxation) of the epithelial layer. All data is expressed as mean ± SEM. α-LTX data is presented as a % of the ACh maximum response and FLRT3 data is presented as %KPSS response.

Table S1. Demographics of the individual patients from whom samples were obtained

No.	Diagnosis	Age	Gender	Samples	FEV1% predicted	Experiments where used
1	Non diseased donor	31	F	IASMC	105	6,7,8
2	Non diseased donor	23	M	IASMC	82	7,8
3	Non diseased donor	22	F	IASMC	87	6,7,8
4	Asthma	39	M	IASMC	84	6,7,8
5	Asthma	29	M	IASMC	89	6,7,8
6	Asthma	21	M	IASMC	108	6,7

7	Asthma	31	M	IASMC	85	6,7
8	Asthma	27	F	IASMC	78	7,8
9	Asthma	33	M	IASMC	78	6,7,8
10	Non diseased donor	69	M	IASMC	N	7,8
11	Non diseased donor	48	M	IASMC	N	7
12	Non diseased donor	22	F	IASMC	N	7
13	Asthma	38	M	Bronchoscopy	72	2
14	NSCCa	68	M	Resection	82	2
15	NSCCa	71	M	Resection	79	2
16	Asthma	33	F	Transplant	N	1
17	Asthma	33	M	Bronchoscopy	N	3
18	Asthma	19	F	Bronchoscopy	N	3
19	Asthma	19	M	Bronchoscopy	N	1,3
20	Asthma	19	M	Bronchoscopy	N	3
21	Asthma	30	M	Bronchoscopy	N	3
22	Asthma	71	M	Resection	N	3
z23	Asthma	22	M	Bronchoscopy	N	1,3
24	Asthma	69	M	Bronchoscopy	N	3
25	Asthma	59	F	Bronchoscopy	N	3
26	Asthma	56	F	Bronchoscopy	85	3
27	Asthma	42	M	Resection	N	3
28	Asthma	21	M	Bronchoscopy	67	2,3
29	Asthma	39	M	Bronchoscopy	N	3
30	Asthma	27	F	Bronchoscopy	79	3
31	Asthma	23	M	Bronchoscopy	82	3
32	Asthma	51	F	Transplant	36	3
33	Non diseased donor	20	M	Bronchoscopy	N	3
34	Non diseased donor	27	F	Bronchoscopy	N	3
35	Non diseased donor	21	F	Bronchoscopy	N	1,3
36	Non diseased donor	30	M	Bronchoscopy	N	3
37	Non diseased donor	31	M	Bronchoscopy	N	1,3
38	Non diseased donor	22	M	Bronchoscopy	N	1,3
39	Asthma/ Alpha 1 antitrypsin	39	M	Transplant	8	5
40	Ca	58	F	Resection	N	5
41	Asthma Death	15	M	Transplant	N	5
42	Asthma	80	M	Bronchoscopy	N	5
43	Non diseased donor	NA	NA	Transplant	N	5
44	Non diseased MVA	42	M	Transplant	N	5
45	Non diseased MVA	NA	NA	Transplant	N	5
46	Non diseased MVA	16	M	Transplant	N	5

	Non diseased			Transplant	N	
47	Hemorrhage	52	M			5
48	Non diseased MVA	25	M	Transplant	N	5
49	Non diseased Hemorrhage	50	M	Transplant	N	5
50	Asthma	17	M	Transplant	N	5
51	Non diseased Trauma	40	M	Transplant	N	5
52	Ca	70	M	Resection	N	5
53	Non diseased donor	30	M	Transplant	N	5
54	Non diseased donor	53	M	Transplant	N	5
55	Asthma Death	15	M	Transplant	N	5
56	Asthma Death	80	M	Transplant	N	5
57	Asthma Death	15	M	Transplant	N	5
58	Non diseased donor	30	M	Transplant	N	5

Abbreviations: M = male, F = female, Ca= carcinoma, NSCCa= non-small cell carcinoma, MVA= motor vehicle accident= HuGene1_0-st-v1 microarray, 2= long range PCR and sequencing, 3= qPCR (LPHN1,2 and 3), 4= LPHN3 Western Immunoblot, 5= LPHN3 Immunohistochemistry, 6= Attachment, 7= Proliferation and 8=ERK1/2 and AKT phosphorylation

Table S2. Top 10 genes up-regulated in asthmatic ASMC compared to healthy ASMC

Gene Symbol	Gene Name	Log2 Fold change	p value	FDR
PDE1C	phosphodiesterase 1C, calmodulin-dependent 70kDa	2.138	1.676E-04	0.257
TPM1	tropomyosin 1 (alpha)	1.234	2.056E-04	0.260
KIAA1324L	KIAA1324-like	2.135	3.126E-04	0.325
PRUNE2	prune homolog 2 (Drosophila)	2.559	5.883E-04	0.384
CYR61	cysteine-rich, angiogenic inducer, 61	1.508	6.068E-04	0.384
ID4	inhibitor of DNA binding 4, dominant negative helix-loop-helix protein	1.546	6.638E-04	0.384
CSRP1	cysteine and glycine-rich protein 1	1.320	7.117E-04	0.384
FAT3	FAT atypical cadherin 3	1.068	7.399E-04	0.384
**LPHN3**	**latrophilin 3**	**2.110**	8.485E-04	0.395
PRUNE2	prune homolog 2 (Drosophila)	2.774	1.002E-03	0.405

Table S3. Top 10 genes down-regulated in asthmatic ASMC compared to healthy ASMC

Gene Symbol	Gene Name	Log2 Fold change	p value	FDR
PLEKHG1	pleckstrin homology domain containing, family G (with RhoGef domain) member 1	-2.260	9.630E-07	0.019
PSG4	pregnancy specific beta-1-glycoprotein 4	-3.524	1.320E-06	0.019
TCN2	transcobalamin II	-1.582	2.990E-06	0.029
PTDSS1	phosphatidylserine synthase 1	-1.043	5.020E-06	0.037
EYA1	eyes absent homolog 1 (Drosophila)	-2.009	1.270E-05	0.066
MLPH	melanophilin	-1.984	2.930E-05	0.122
GALNT5	UDP-N-acetyl-alpha-D-galactosamine:polypeptide N-acetylgalactosaminyltransferase 5 (GalNAc-T5)	-1.425	4.410E-05	0.143
IGDCC4	immunoglobulin superfamily, DCC subclass, member 4	-1.920	5.470E-05	0.159
PARD6G	par-6 family cell polarity regulator gamma	-1.276	6.960E-05	0.184
PTPRU	protein tyrosine phosphatase, receptor type, U	-1.245	8.660E-05	0.210

Table S4. Gene Set Enrichment Analysis (GSEA)

NAME	FDR q-val
**Enriched for genes up-regulated in asthmatic ASMC relative to healthy controls**	
REGULATION OF MUSCLE CONTRACTION	3.93E-02
POSITIVE REGULATION OF BINDING	4.10E-02
REGULATION OF HEART CONTRACTION	4.55E-02
**Enriched for genes down-regulated in asthmatic ASMC relative to healthy controls**	
GLYCOLIPID METABOLIC PROCESS	5.80E-03
JAK STAT CASCADE	8.49E-03
LIPID BIOSYNTHETIC PROCESS	1.27E-02
LIPID METABOLIC PROCESS	2.31E-02

Table S5. SNPs for *LPHN1* and *LPHN3* associations with severity of BHR within asthma

CHR	SNP	BP	TA	BETA	L95	U95	P value	FDR
LPHN1								
19	rs3810256	14120846	A	-0.011	-0.290	0.268	0.941	1
19	rs2420416	14159442	G	-0.095	-0.292	0.101	0.342	1
LPHN3								
4	rs12509742	62327627	A	0.023	-0.145	0.192	0.787	1
4	rs2345043	62330503	T	0.023	-0.145	0.192	0.787	1
4	rs1450903	62352873	G	-0.005	-0.278	0.269	0.973	1
4	rs10517547	62354238	A	0.121	-0.113	0.355	0.310	1
4	rs11734607	62376287	T	-0.003	-0.183	0.178	0.977	1
4	rs2345041	62380952	A	-0.095	-0.316	0.126	0.398	1
4	rs2015569	62401922	T	0.033	-0.246	0.311	0.818	1
4	rs7667328	62409295	G	0.103	-0.192	0.398	0.496	1
4	rs10446786	62409469	G	-0.076	-0.261	0.109	0.421	1
4	rs13110933	62416032	C	-0.047	-0.223	0.129	0.599	1
4	rs6551665	62422136	G	-0.116	-0.291	0.058	0.191	1
4	rs6846033	62422269	C	0.093	-0.121	0.306	0.394	1
4	rs9683662	62430979	T	0.000	-0.190	0.191	0.996	1
4	rs6858066	62436915	A	-0.052	-0.226	0.123	0.561	1
4	rs11131347	62441865	T	-0.030	-0.200	0.141	0.734	1
4	rs1470724	62444465	C	0.062	-0.130	0.254	0.526	1
4	rs6551666	62446070	C	-0.056	-0.256	0.143	0.580	1
4	rs10517549	62471863	G	0.008	-0.182	0.198	0.932	1
4	rs734644	62483323	T	-0.009	-0.209	0.192	0.933	1

4	rs1450896	62499334	A	0.123	-0.413	0.659	0.653	1
4	rs995447	62501063	C	-0.130	-0.452	0.191	0.427	1
4	rs1510925	62513128	G	-0.033	-0.250	0.184	0.765	1
4	rs1397545	62517697	A	-0.021	-0.299	0.256	0.880	1
4	rs1397543	62521660	T	-0.037	-0.256	0.182	0.742	1
4	rs1397548	62528085	A	-0.068	-0.260	0.123	0.485	1
4	rs17292128	62533752	C	0.066	-0.159	0.291	0.566	1
4	rs10017760	62539254	A	-0.159	-0.384	0.066	0.166	1
4	rs2271339	62544980	G	0.174	-0.022	0.370	0.082	1
4	rs13115125	62550048	G	-0.026	-0.197	0.145	0.764	1
4	rs1510920	62566026	C	0.127	-0.289	0.542	0.549	1
4	rs6827266	62584757	T	-0.010	-0.177	0.158	0.909	1
4	rs1397546	62597619	C	0.007	-0.164	0.178	0.936	1
4	rs11736888	62606392	T	0.053	-0.154	0.259	0.617	1

Abbreviations used CHR= Chromosome, BP = base pair, TA=tested allele, U95=95% Upper limit, L95=95% Lower limit

References

1. Dijkstra AE, Postma DS, van Ginneken B, et al. Novel genes for airway wall thickness identified with combined genome-wide association and expression analyses. American journal of respiratory and critical care medicine 2015;**191**(5):547-56.
2. Gosens R, Stelmack GL, Dueck G, et al. Role of caveolin-1 in p42/p44 MAP kinase activation and proliferation of human airway smooth muscle. American Journal of Physiology-Lung Cellular and Molecular Physiology 2006;**291**(3):L523-L34.
3. Johnson PR, Burgess JK, Underwood PA, et al. Extracellular matrix proteins modulate asthmatic airway smooth muscle cell proliferation via an autocrine mechanism. Journal of Allergy and Clinical Immunology 2004;**113**(4):690-96.
4. Donovan C, Royce SG, Esposito J, et al. Differential effects of allergen challenge on large and small airway reactivity in mice. PloS one 2013;**8**(9):e74101.
